# The Effects of COVID-19 Vaccination on Lactating Women: A Systematic Review of the Literature

**DOI:** 10.3389/fimmu.2022.852928

**Published:** 2022-04-08

**Authors:** Joke Muyldermans, Louise De Weerdt, Larissa De Brabandere, Kirsten Maertens, Eline Tommelein

**Affiliations:** ^1^ Department of Pharmaceutical Sciences (FARM), Faculty of Medicine and Pharmacy, Vrije Universiteit Brussel, Jette, Belgium; ^2^ Midwifery Education, Department of Health, University College Brussels, Jette, Belgium; ^3^ Centre for the Evaluation of Vaccination, Vaccine and Infectious Diseases Institute, University of Antwerp, Antwerp, Belgium

**Keywords:** COVID-19, obstetrics, immunology, breastfeeding, vaccination, lactation

## Abstract

**Objectives:**

The availability of new vaccines against COVID-19 urges for guidance about vaccination during lactation. We aimed to review the literature to get an insight into the effects of COVID-19 vaccination on lactating women.

**Design:**

Systematic review.

**Data Sources:**

We searched Ovid Embase Classic+Embase, PubMed and BioMed Central for articles published between December 1^st^ 2020 and December 31^st^ 2021.

**Review Methods:**

The search strategy contained terms and combinations related to COVID-19 vaccination during lactation, including the MeSH terms “COVID-19”, “COVID-19 Vaccines”, “SARS-CoV-2”, “Lactation”, “Breast Feeding”, “Pregnancy” and “Postpartum period”. The database search was completed with a manual search of the reference lists of included articles. Data concerning country, study period, number of participants, type of applied vaccine, time points of sampling and outcome measures were collected from the selected manuscripts. The data are summarized and synthesized in a descriptive way.

**Results:**

30 manuscripts were included in this review. Data on safety of COVID-19 vaccination during lactation indicate no severe vaccine-related local and systemic reactions, both after first and second dose, neither in the mother nor the nursing child. No significant amount of vaccine components seems to appear in breast milk. Milk supply data after vaccination are inconclusive as there are no quantitative data available. Some women however observe a temporary increase or reduction in milk supply, without long-term effects. All prospective cohort studies demonstrated the presence of SARS-CoV-2-specific antibodies in breast milk of nursing mothers vaccinated against SARS-CoV-2. Nearly all studies were conducted with mRNA vaccines.

**Conclusion:**

There is evidence that the administration of a COVID-19 vaccine is safe and poses no additional risk to the breastfeeding woman or the breastfed baby. After vaccination of the mother during the lactation period, antibodies appear in the milk, which could protect the infant against COVID-19. Professional associations and government health authorities should therefore recommend offering COVID-19 vaccines to breastfeeding women, as the potential benefits of maternal vaccination while breastfeeding outweigh the risks.

## Highlights

What is already known on this topic

- Healthcare professionals are at greater risk to get a COVID-19 infection
- It was recommended to prioritize healthcare professionals for vaccination with the new vaccines against COVID-19
- Many healthcare professionals are women and at fertile age, who are possibly breastfeeding
- Exclusively breastfeeding for six months and after that for two years in combination with complementary foods is recommended
- None of the COVID-19 vaccines currently authorized or in phase 3 have been trialed for women who are breastfeeding

What this study adds

- COVID-19 vaccines during breastfeeding pose no risk to the woman and infant
- The presence of antibodies in breast milk of nursing mothers after COVID-19 vaccination was demonstrated
- No significant amounts of COVID-19 vaccine components were found in breast milk after vaccination
- Some women report a temporary milk supply change after COVID-19 vaccination

## Introduction

The COVID-19 outbreak was characterized as a pandemic in March 2020 by the World Health Organization (WHO) (1). SARS-CoV-2, the virus that causes COVID-19, appears with a variety of clinical manifestations. In most cases, the disease starts with influenza-like symptoms and evolves in some patients towards acute respiratory distress syndrome (ARDS) and pneumonia. However, other symptoms like, gastrointestinal, dermatological, neurological, cardiovascular, and renal manifestations have also been reported (2). Breastfeeding women can, similar to other populations, get infected by the SARS-CoV-2-virus.

In October 2020, the European Commission listed a number of key steps for effective vaccination strategies ensuring access to safe vaccines across Europe (3). By the end of 2020, a worldwide vaccination strategy deployed. In multiple countries, people in residential centers, along with health care providers (HCPs) were prioritized for vaccination. At the start of the vaccination strategy, mRNA-based, adenovector-based vaccines, inactivated whole virus and subunit vaccines were approved and used (4, 5).

The availability of new vaccines against COVID-19 and the recommendation to prioritize HCPs for vaccination, urged for guidance about vaccination during lactation as many of these HCPs are of fertile age. Initially, multiple governmental guidelines advised against vaccination during the lactation period, disregarding a breastfeeding woman’s likelihood of developing a severe form of the COVID-19 after exposure to the virus. At a later stage, the vaccination campaign was extended to the general population, including women of fertile age. This led to discontent and a firm counter reaction in the scientific literature (5, 6). The Center for Disease Control and Prevention (CDC) (7), the European Medicines Agency (EMA) (8) and the Royal College of Obstetricians and Gynaecologists (RCOG) (9) have since reversed their stand and now advise to offer the vaccine to breastfeeding women (10). Although the currently marketed COVID-19 vaccines are non-replicating vaccines and therefore theoretically pose no risk (9, 11, 12), several institutions still mention that robust safety and immunogenicity data in this population of women is lacking.

Indeed, none of the COVID-19 vaccines currently authorized or in phase 3 have been trialed for women who are breastfeeding. Some of these vaccines are now being tested in an academic setting. As vaccination in the postpartum period and during lactation could however result in clinically relevant immunologic factors in breast milk that are protecting the child in early life, it is of importance that women have this information to decide whether to take the vaccine. Exclusively breastfeeding for the first six months, and after six months in combination with complementary foods, is recommended by the World Health Organization (13) and UNICEF (14), given the many physically and psychologically benefits of breastfeeding, for both mother and child. Risking a mother to stop breastfeeding earlier than intended because of vaccination, should be regarded as a threat to the health of both. On the other hand, refusing vaccination also entails risks for the mother, with an increased risk of infection and development of (severe) COVID-19.

We aim to review the literature to get an insight in the effects of vaccination with COVID-19 vaccines during the lactation period. This entails the safety of vaccination during lactation, the immune response in lactating women and the excretion of immunological factors in breast milk. Neonates rely on the transfer of immunity *via* the placenta and breast milk, since they are born with an immature immune system. The role of immunoglobulin G (IgG) transferred *via* the placenta is well established, but less is known about the transfer of antibodies and the mechanisms by which these antibodies provide protection to the neonate *via* breast milk (15). It is therefore plausible that the immune response triggered by vaccinating lactating women may be different from the general population. The conclusions will contribute to the knowledge on COVID-19 vaccination and the results will benefit the population with respect to public health.

## Methods

### Study Design and Searches

We searched Ovid Embase Classic+Embase, PubMed and BioMed Central for articles published between December 1^st^ 2020 and December 31^st^ 2021. The search strategy contained terms and combinations related to COVID-19 vaccination during lactation, including the MeSH terms “COVID-19”, “COVID-19 Vaccines”, “SARS-CoV-2”, “Lactation”, “Breast Feeding”, “Pregnancy” and “Postpartum Period”. The final literature search was performed on the 31^st^ of December 2021. The database search was completed with a manual search of the reference lists of included articles (i.e. “snowballing”). The quality of reporting was supported by the use of the PRISMA guidelines (16). The detailed overview of the search strategy is provided in **Appendix 1**.

### Eligibility Criteria and Study Selection

Manuscripts were eligible for inclusion if (1) study participants were women vaccinated with a COVID-19 vaccine during the lactation period and (2) the results reported on the safety of vaccination during lactation OR on the excretion of COVID-19 vaccine components in breast milk OR the excretion of immunological factors in breast milk after COVID-19 vaccination OR on the impact of COVID-19 vaccination on the breast milk production. Manuscripts published in English, French, Dutch, German or Spanish were included. Studies that focused on women with specific pathologies (e.g. transplant patients) were excluded. JM and ET independently screened titles, abstracts and full-texts of the retrieved manuscripts for eligibility. Each manuscript showing uncertainty regarding inclusion criteria was discussed with the other authors until consensus about inclusion.

### Data Collection, Synthesis and Analysis

Data concerning country, study period, number of participants, type of applied vaccine, time points of sampling and outcome measures were collected from the selected manuscripts. The datasets were summarized and synthesized in a descriptive way.

## Results

### Included Studies

**Figure 1:**
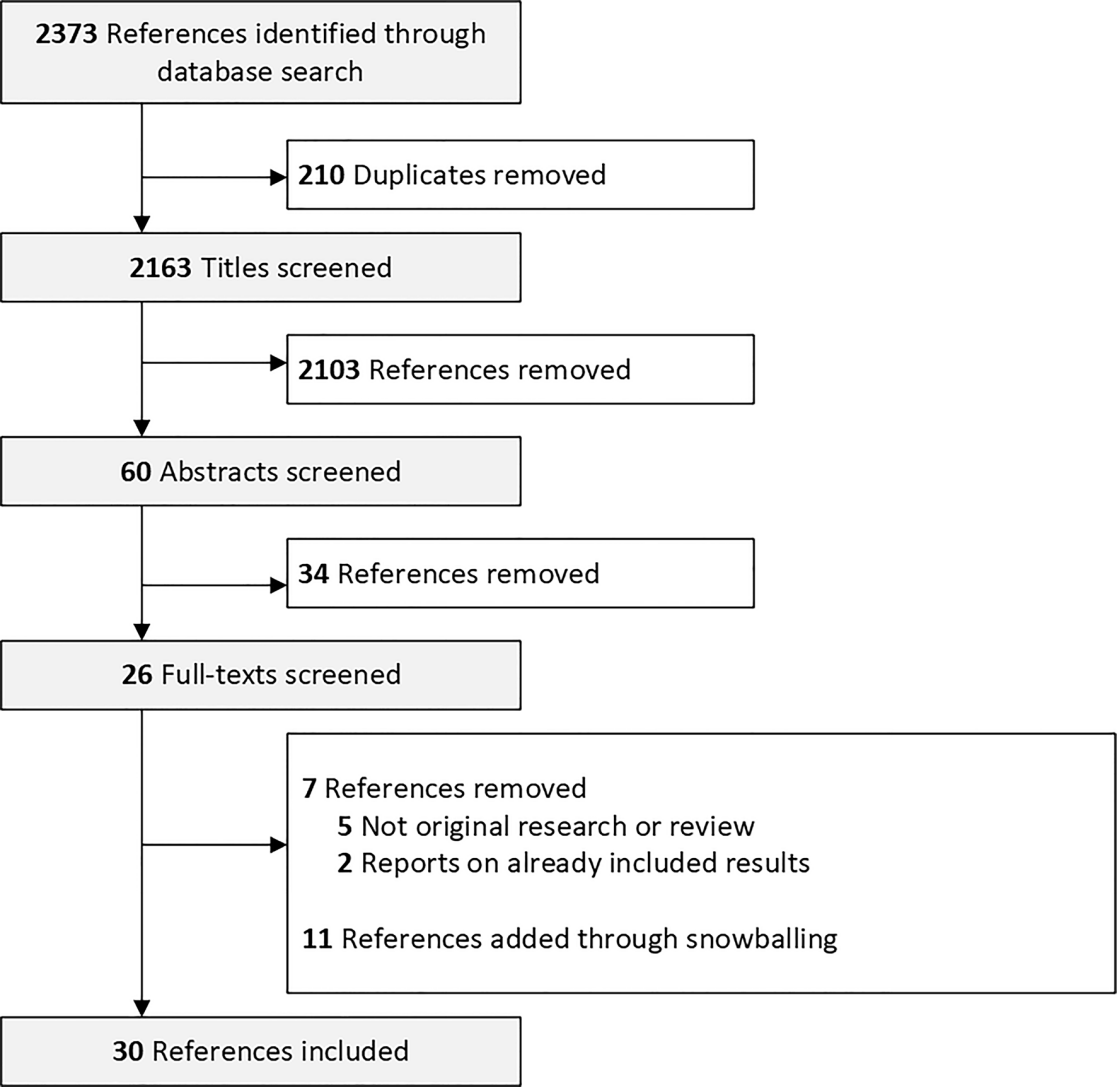
Flow diagram of literature search and manuscript selection.

In Total, 2,373 manuscripts were identified. We screened 2,163 titles and 60 abstracts for eligibility. We screened the full text of 26 manuscripts and excluded 7. Eleven manuscripts were added *via* manual search of the references (**Figure 1**). We included manuscripts reporting on women that were vaccinated with the messenger RNA (mRNA) mRNA-1273 (Moderna) and vaccines BNT162b2 (Pfizer–BioNTech), the adenoviral vector vaccines ChAdOx1 nCoV-19 (Oxford - AstraZeneca) and JNJ-78436735 (Johnson & Johnson) and the inactivated whole-virus SARS-CoV-2 vaccine by Sinovac Biotech Ltd.

Of the 30 included references, 1 study reports on the excretion of COVID-19 components in breast milk (17), 20 studies report on the excretion of antibodies in breast milk (18–36), 1 study report on the excretion of other immunological factors (37) and 1 study reports on the impact of vaccination on breast milk production (38). Two studies report on both side effects and the excretion of COVID-19 antibodies (39, 40), 1 study reports on the excretion of COVID-19 antibodies and the excretion of vaccine components (41), 3 studies report on side effects and the impact of vaccination on breast milk production (42–44) and 1 study reports on side effects, the impact of vaccination on breast milk production and on breast milk production (45).

### Side-Effects in the Mother After COVID-19 Vaccination During Lactation

Six publications report on side-effects of COVID-19 vaccination during lactation in a total of 7,241 women, of which 4,509 vaccinated with the BNT162b2 vaccine, 2,669 with the mRNA-1273 vaccine and 23 with the JNJ-78436735 vaccine (39, 42–45).

In a first study (42), data from 17,525 women vaccinated with a COVID-19 vaccine were included of which 6,815 were lactating women. The other women were either pregnant women (n = 7,809) or women from fertile age planning to get pregnant at the moment of the first vaccine dose (n = 2,901). Of the vaccinated lactating women, 4,156 received the BNT162b2 vaccine, 2,596 received the mRNA-1273 vaccine, 23 received the JNJ-78436735 vaccine and from 40 women it was not known which vaccine they received. The most common adverse reactions in all participating women after a first vaccine dose were pain at the injection site (16,019/17,525; 91.4%) and fatigue (5,489/17,525; 31.3%). Fatigue was more reported after the second dose (10,399/17,525; 69.2%). No difference in the rate of adverse events by vaccine-type was reported across all groups (42).

Another study (43) (n = 180, of which 128 lactating women received the BNT162b2 vaccine and 52 the mRNA-1273) reported similar proportions of general side-effects for both the BNT162b2 (89.4%) and mRNA-1273 (98.1%) vaccine after the first dose in lactating women. The most common reactions in lactating women after a first dose were pain at the injection site (105/126; 86.8% BNT162b; 50/52, 96.2% mRNA-1273) fatigue (31/126; 26.3% BNT162b2; 12/52; 23.1% mRNA-1273) and headache (28/126; 23.7% BNT162b;13/52; 25.0% mRNA-1273). Following the second dose, lactating women receiving the mRNA-1273 vaccine reported significantly more side-effects like chills (36/52 vs. 55/123, 75.0% vs.47.8%), muscle/body aches (41/52 vs. 71/123, 83.7% vs.61.7%), fever (23/52 vs. 28/123, 46.9% vs. 24.3%) and vomiting (4/32 vs. 1/123, 8.5% vs. 0.9%) (p<0.05) in comparison with women receiving the BNT162b2 vaccine. Local symptoms including redness at injection site (15/52 vs. 3/123, 31.9% vs. 2.6%), swelling at injection site (14/52 vs. 8/123, 29.8% vs. 6.1%) and itching at injection site (8/52 vs. 5/123, 17.4% vs. 4.4%) were more common after the second dose with the mRNA-1273 vaccine (p<0.05) then after the BNT162b2 vaccine (43).

In a third study (45), (n = 48), fever, chills, muscle or body aches, fatigue and/or tiredness and joint pain were significantly less reported by lactating women after the first dose than after the second dose. All 21 participants (100%) who received the mRNA-1237 vaccine reported symptoms at injection, while only 21 (78%) of 27 BNT162b2 participants reported symptoms at injection site (p=0.02). During the study, two infants were diagnosed with COVID-19. One week after the second dose, mild symptoms by one infant were reported, while the infant’s PCR test was positive, the mother who was vaccinated had a negative test. A positive plasma anti-SARS-CoV-2 IgG (immunoglobulin G) and IgA (immunoglobulin A) was found in another infant, despite the mother reported no known prior SARS-CoV-2 infection and receiving the vaccine postpartum. A likely natural asymptomatic COVID-19 infection could be indicated, since antibodies against SARS-CoV-2 nucleocapsid protein were found in the mother’s plasma (45).

A fourth study (39) (n = 84) reported similar adverse reactions after the first and second dose in lactating women. Forty-seven women (55.9%) reported one or more vaccine-related side effect after the first dose of which 40 local pain at injection site (47.6%), and 8 fatigue (9.5%). After the second dose, 52 women (61.9%) reported side-effects, of which 34 local pain at injection site (40.5%), 28 fatigue (33.3%) and 10 fever (11.9%) (39). In the final included study (44), local reactions at the injection site (redness, pain or swelling) were reported by 57 of 88 women (64.8%), headache, muscle pain or joint pain was reported by 52/88 (59.1%) and fatigue by 54/88 (61.4%). Five out of 88 (5.7%) lactating women reported neck or axillary lymph node swelling after their second dose of the BNT162b2 vaccine. Mastitis was reported by three (3.4%) women and breast engorgement which resolved after 24 hours was reported by one woman (44).

In a fifth study (40) (n = 26) one or more side effects were reported by 57% of the participants after the first dose. After the second dose, one or more side effects were reported by 81% of the participants. After the first dose, the most reported side effects were local pain or swelling (6/26, 28.6%) and muscles aches (5/26, 23.8%). After the second dose fatigue (7/26, 33.3%), local pain or swelling (6/26, 28.6%), fever (5/26, 23.8%) and headache (5/26, 23.8%) were the most frequently reported side effects (40).

Overall, we can conclude that side-effects in lactating mothers after COVID-19 vaccination are similar to other individuals like pregnant women and women who are planning to get pregnant (42). The most common side-effects shown in these studies were fatigue, fever, headache, chills, muscle pain and pain at injection site. These side-effects are mild and similar to side-effects described in the general population (46). Studies showed increased reactions following the second dose of BNT162b2 and mRNA-1237 vaccines compared to the first dose (39, 42, 43, 45).

### Side-Effects in the Infant After COVID-19 Vaccination During Lactation

Three studies evaluated side-effects in the infant after a COVID-19 vaccination to the mother, including a total of 7,043 infants (4,311 of whom the mother was vaccinated with the BNT162b2 vaccine, 2,669 with the mRNA-1273 vaccine, 23 with the JNJ-78436735 vaccine, 40 of which the vaccine type was not specified) (42, 43, 45).

In the first study (n = 6,815) 208 breastfeeding mothers reported to have concerns about the infant after the first dose (3.0%) and concerns were reported by 267 breastfeeding mothers after the second dose (4.4%) (42). In another study (n = 180) the most common side-effects seen in the nursing children were similar for both mRNA vaccines following the first dose (poor sleep: 4/129, 3.4% BNT162b2; 3/53, 5.9% mRNA-1273 and irritability: 2/129, 1.7% BNT162b2; 2/53, 3.9% mRNA-1273) and second dose (poor sleep: 7.8% BNT162b2; 8.3% mRNA-1273 and irritability: 12/126, 10.3% BNT162b2; 5/53, 10.4% mRNA-1273). The only side-effect showing a significant difference between the mRNA-1273 and BNT162b2 vaccine after the second dose was drowsiness after the second vaccine dose. Mothers who received the mRNA-1273 vaccine reported significantly more drowsiness in the infant after the second dose in comparison to mothers who received the BNT162b2 vaccine (3/53 vs. 0/129; 6.4% vs. 0.0%; p=0.02) (43). In another study with 48 participants (27 BNT162b2 and 21 mRNA-1273), 12% (6/48) of mothers reported to have seen at least one symptom after the first vaccine in their infants. These included gastrointestinal (2/48; 4.0%; BNT162b2), sleep changes (3/48; 6.0%; 2 BNT162b2, 1 mRNA-1273) and rash/baby acne (1/48; 2.0%; mRNA-1273). No mothers reported an infant symptom after the second vaccine dose (45).

### Excretion of COVID-19-Vaccine Components in Breast Milk

Two manuscripts report on the excretion of COVID-19 vaccine particles in breast milk, including a total of 16 women (15 vaccinated with the BNT162b2 vaccine and 1 with the mRNA-1273 vaccine) (17, 41).

The first study included 6 women receiving a COVID-19 vaccine of which 5 women were vaccinated with the BNT162b2 vaccine, and 1 with the mRNA-1273 vaccine. When testing breast milk samples, no mRNA was found in their milk 4-48 hours post-vaccination (17). The second study included 10 women receiving the BNT162b2 vaccine of which milk samples were analyzed at four timepoints (pre-vaccination, 1-3 days after the first dose, 7-days after the first dose and 3-7 days after the second dose). Across all timepoints, a minimal transfer of BNT162b2 mRNA in human milk was found. Very low levels of vaccine mRNA were only found on rare occasions (4/40) and this within the first week after the first dose or second dose. Detectable levels of vaccine mRNA were not shown in 90% (36/40 samples) of the samples. The highest concentration of BNT162b2 mRNA found was 2 ng/mL. In the worst-case scenario, this would mean 0.67% of the given vaccine dose being transferred to the infant in 100 mL of breast milk (41).

mRNA vaccines contain a part of the genetic code of the SARS-Cov-2 virus, more specifically that of the SARS-Cov-2 S spike or “S” protein. They are encapsulated in very small specialized particles consisting of fats, cholesterol and polyethylene glycol. The small amount of polyethylene glycol-2000 in the BNT162b2 vaccine was also not found in breast milk (45).

### Excretion of Antibodies in Breast Milk

The eventual list of included manuscripts covering the excretion of antibodies in breast milk is presented in **Tables 1**, **2**. It covers an overview of the number of participants, applied vaccines, timepoints of breast milk collection and IgG and IgA responses in breast milk for every study. Our final sample comprised of 18 published (18, 19, 21–33, 39, 40) manuscripts reporting on 716 women receiving a COVID-19 vaccine during the lactation period and 6 preprints (20, 35, 36, 41, 45, 48) on 151 women receiving a COVID-19 vaccine during the lactation period. In total, 665 women were vaccinated with the BNT162b2 vaccine, 125 with the mRNA-1273, 13 with the JNJ-78436735, 44 with the ChAdOx1 nCoV-19 and 20 with the Sinovac Biotech Ltd. The studies were performed in 9 different countries (i.e. USA, Israel, Spain, Brazil, Italy, Poland, Portugal, Singapore and The Netherlands).

**Table 1:** Overview of vaccination studies in breastfeeding women with data on antibody secretion in breast milk.

Study	No. of participants	Applied vaccine(s)	Time points	IgA response in BM	IgG response in BM
Baird et al. (21) *(USA)*	6	BNT162b2 (n = 3)	Before vaccination (BV) + day 1, 4, 7, 11, 14 after 1^st^ dose + 1 day before 2^nd^ dose + day 1, 4, 7, 11 and 14 after 2^nd^ dose	Elevated levels of IgA beginning at day 7 after 1^st^ dose. Prior to the 2^nd^ dose, levels of IgA decreased. Levels of IgA increased sharply after 2^nd^ dose.	Elevated levels of IgG after 1^st^ dose, beginning at day 7, with an IgG dominant response. The level of IgG decreased prior to 2^nd^ dose. IgG levels sharply increased after 2^nd^ dose.
		mRNA-1273 (n = 3)			
Calil et al. (22) *(Brazil)*	20	Sinovac Biotech Ltd. (inactivated whole-virus SARS-CoV-2 vaccine)	BV + Weekly after 2^nd^ dose for 3 weeks + until 4 months after 1^st^ dose (n=10)	After the 1^st^ dose, mean levels of IgA increased in the first two weeks. At week 5 and 6, significantly higher mean values were obtained compared to week 1, 2, 3 and 4. At week 7, specific IgA antibody levels above the seroconversion were found in milk samples of 10 mothers. IgA levels were above the seroconversion in milk samples 4 months after the 1^st^ dose (n=10).	Not Applicable
Charepe et al. (23) *(Portugal))*	14	BNT162b2	1-3 weeks after 1^st^ dose + 1-3 weeks after 2^nd^ dose	IgA was detected in breast milk after vaccine administration. In 35.7% (5/14) of milk samples, IgA was present after the 1^st^ dose. IgA was present in 21.4% (3/14) after the 2^nd^ dose.	IgG was detected in breast milk was detected after vaccine administration. After the 1^st^ dose, IgG was present in 7.1% (1/14). IgG presence increased to 42.9% (6/14) after the 2^nd^ dose.
Collier et al. (24) (*USA)*	16	mRNA-1273 (n = 5)	Close to each vaccine dose and between 2-8 weeks after 2^nd^ dose	The median IgA titer was 25 after vaccination.	The median IgG titer was 97 after vaccination.
		BNT162b2 (n = 11)			
Esteve-Palau et al. (25) (*Spain)*	33	BNT162b2	Around 2 weeks after 1^st^ dose (T1) + 2 (T2) and 4 weeks (T3) after 2^nd^ dose	Not Applicable	Median IgG levels for breast milk were found at each time point: 1 (0-2.9) AU/mL for T1, 78 (33.7-128) AU/mL for T2, and 50.4 (24.3-104) AU/mL for T3.
Gray et al. (26) *(USA)*	31	mRNA-1273 (n = 15)	BV (T1) + Day of 2^nd^ dose (T2) + between 2-6 weeks after 2^nd^ dose (T3)	in milk samples after mRNA-1273 vaccination, higher S- and RBD-specific IgA responses were found compared to the BNT162b2 vaccine. There was no significant rise in IgA after either dose.	from T1 to T3 IgG rose significantly (3.44-3.50; p=0.002), but not from T1 to T2 (3.44-3.45, p=0.7).
		BNT162b2 (n = 16)			
Guida et al. (27) *(Italy)*	10	BNT162b2	20 days after 1^st^ dose (T1) (before 2^nd^ dose) + 7 days after 2^nd^ dose (T2)	Not Applicable	Anti-SARS-CoV-2 S antibodies were detected in two (40%) milk samples with a low concentration (1.2 +/- 0.3 U/mL) at T1. In all milk samples anti-SARS-CoV-2 S antibodies were detected at T2 (41.5 +/- 47.5 U/mL).
Jakuszko et al. (28) *(Poland)*	28	BNT162b2	Day 8 and 21 after 1^st^ dose (day 21 prior to 2^nd^ dose) + Day 29, 43 after 2^nd^ dose	No differences in the absolute values were observed on day 8. On day 29 after the 2^nd^ dose, the highest concentrations of IgA were observed, with a decrease on day 43.	In the absolute values, there were no differences observed on day 8. In 14/28 (50%) positive IgG samples were observed on day 22 and in all women on days 29 and 43. On day 29 after the 2^nd^ dose the highest concentrations of IgG were observed. A decrease was seen on day 43.
Juncker et al. (40) *(The Netherlands)*	26	BNT162b2 (6 one dose, 20 2 doses)	BV + Day 3, 5, 7, 9, 11, 13 and 15-17 after 1^st^ dose + before 2^nd^ dose + Day 3, 5, 7, 9, 11, 13 and 15-17 after 2^nd^ dose	After vaccination, a higher inter-individual variability in IgA was observed. IgA started rising 5 to 7 days after 1^st^ dose, with an increase of 12% per day. On day 15 a three-fold increase was seen, compared to baseline. From day 15 after 1^st^ dose and just before 2^nd^ dose, IgA levels decreased by 43%. IgA levels stabilized at 50% of peak level. At 2^nd^ dose peak level was 1.3 times higher compared to peak level 7 days after 1^st^ dose. After the 2^nd^ dose IgA gradually declined, decreasing by 33% until the end of sample collection 35 days after 1^st^ dose IgA increased by 2.4 times.	Not Applicable
Kelly et al. (18) *(USA)*	5	BNT162b2	BV + Day of 1^st^ dose + weekly following until between 40-90 days after 1^st^ dose	In all samples, IgA levels were elevated compared to pre-vaccine baseline. Two weeks after the 1^st^ dose, IgA remained sustained. Following the 2^nd^ dose gradual decline in IgA over time was seen.	In all samples, IgG levels were elevated relative compared to pre-vaccine baseline. Starting at 20 days after 1^st^ dose, IgG remained sustained at an elevation through final milk sample.
Lechosa-Muñiz et al. (19) *(Spain)*	110	BNT162b2 (n = 70)	30 days after 2^nd^ dose for BNT162b2 and mRNA-1273	According to the type of vaccine, the mean IgA titers observed were different. Mothers who receveid the BNT162b2 vaccine had a mean of 0.11 (AU), for mRNA-1273 the mean was 0.10 (AU) and for ChAdOx1-S (one dose) the mean was 0.04 (AU). Comparing mean of IgA in mother milk from mothers vaccinated with BNT162b2 vs. ChAdOx1-S, there were significant differences found.	According to the type of vaccine, the mean IgG titers observed were different. Mothers who received the BNT162b2 vaccine had a mean of 0.41 (AU), for mRNA-1273 the mean was 0.45 (AU) and for ChAdOx1-S (one dose) the mean was 0.09 (AU). Comparing mean of IgG in mother milk from mothers vaccinated with BNT162b2 or mRNA-1273 vs. ChAdOx1-S (Sidakmethod), there were significant differences found. No differences in mean IgG could be found between those mothers vaccinated with BNT162b2 vs. mRNA-1273.
		mRNA-1273 (n = 20)	30 days after 1^st^ dose for ChAdOx1-S		
		ChAdOx1-S (n = 20) (only one dose was administrated from ChAdOx1-S)			
Low et al. (29) *(Singapore)*	14	BNT162b2	BV (T1) + 1-3 days after 1^st^ dose (T2) + 7-10 days after 1^st^ dose (T3) + 3-7 days after 2^nd^ dose (T4) + 4-6 weeks after 2^nd^ dose	A strong IgA response at 3-7 days after the 2^nd^ dose (T4) was induced by vaccination. From T4 mother milk samples showed medians of 827 pM of anti-spike and 282 pM of anti-RBD IgA, a significantly higher level compared to the concentrations from earlier time points (p < 0.001). A reduction was observed 4-6 weeks after 2^nd^ dose in the anti-spike (median: 499 pM) and the anti-RBD (median: 0 pM) IgA response.	At 3-7 days after the 2^nd^ dose (T4) the median concentrations of anti-spike and anti-RBD IgG were 392 and 188 pM (picomolar). In all mother milk samples an increase in IgG was observed at T4. At 4-6 weeks after the 2^nd^ dose (T5) the IgG levels remained high, with median concentrations of 657 pM anti-spike IgG and 184 pM anti-RBD IgG. Compared to the IgG concentration the levels at after the 2^nd^ dose (T4, T5) were significantly higher compared to the concentration before vaccination (p < 0.001).
Nir et al. (30) *(Israël)*	64	BNT162b2	During postpartum hospitalization, mean time interval between 2^nd^ dose and delivery was 21.7 (+/- 11.0)	Not Applicable	SARS-CoV-2 IgG was found in all breast milk samples.
Perl et al. (39) *(Israël)*	84	BNT162b2	BV + Weekly for 6 weeks beginning 2 weeks after 1^st^ dose	Mean levels of IgA increased rapidly. At 2 weeks after the 1^st^ dose, mean levels of IgA were significantly elevated, compared to mean levels before vaccination (2.05 ratio; p < 0.001). An increase from 61.8% positive tested samples to 86.1% 1 week after the 2^nd^ dose. Until the last sample, mean levels remained elevated. At 6 weeks, 65.7% of samples tested positive.	The first 3 weeks after vaccination IgG remained low. An increase was seen at week 4 (20.5 U/mL; p = 0.004). At that point 91.7% of samples tested positive, even more increasing to 97% at weeks 5 and 6.
Romero Ramirez et al. (31) *(Spain)*	98	BNT162b2 (n = 92)	14 days after 2^nd^ dose	IgA was found in 89% of the samples (95% CI: 81–95).	Anti- SARS-CoV-2 RBD-S1 IgG in all milk samples of vaccinated mothers. The mean IgG level was 12.19 ± 11.74 BAUs per mL (95% CI: 9.77–14.60; p <.001). The mean IgG levels were significantly higher than the levels from the control group (no vaccination, no previous infection) (0.02 ± 0.05 BAUs per mL [95% CI: 0.01–0.05; p<0.001]).
		mRNA-1273 (n = 6)			
Schwartz et al. (47) *(Israël)*	61	BNT162b2	Time of sample collection according to vaccination was not mentioned in the article	In 15% of mother milk samples IgA was detected in secretory form. A median of 0.4 S/Co (IQR, 0.3e 0.7) was found.	A median igG concentration of 6.3 S/Co (IQR, 5.1e 7.4). was found in all mother milk samples.
Selma-Royo et al. (32) (*Spain)*	75	BNT162b2 (n = 30)	14 days after 1^st^ dose, 14 days after 2^nd^ dose of mRNA vaccines.	IgA had a strong reactivity after the 2^nd^ dose. After the 1^st^ dose, IgA levels were higher in mRNA-1273 vaccinated women compared to ChAdOx1-S vaccinated women (p<0.0001) and BNT162b2 vaccinated women (p=0.002). No differences were found between the two mRNA-based vaccines after the 2^nd^ dose. After the 2^nd^ dose, IgA levels did not further increase.	IgG had a strong reactivity after the 2^nd^ dose. After the 1^st^ dose, higher levels of IgG were induced by the BNT162b2 vaccine and the mRNA-1273 vaccine, compared to the ChAdOx1-S vaccine. The maximum effect with the mRNA-based vaccines was induced 2 weeks after the 2^nd^ dose. A higher percentage of samples from mRNA-based vaccines remained positive compared to ChAdOx1-S 2 weeks after the 1^st^ dose (p<0.0001). After the 1^st^ dose, a higher increment of IgG was shown in mother milk samples from mothers receiving a mRNA vaccine, compared to the ChAdOx1-S vaccine (p<0.0001). IgG levels reached higher levels after the 2nd dose, compared to the 1^st^ dose.
		mRNA-1273 (n =21)			
		ChAdOx1-S (n = 24) (only one dose was administrated from ChAdOx1-S)			
Valcarce et al. (33) *(USA)*	21	BNT162b2 (n = 14)	BV (T1) + 16–30 days after 1^st^ dose (T2) + 7–10 days after 2^nd^ dose (T3)	IgA statistically significantly increased between samples before vaccination (T1) to 16-30 days after the 1^st^ dose (T2) (p < 0.0007) and from T1 to 7-10 days after the 2^nd^ dose (T3) (p < 0.0001). A positive result for SARS-CoV-2 IgA was found in 85% after full vaccination based on the established cutoff value.	All samples were positive for SARS-CoV-2 IgG by 7-10 days after the 2^nd^ dose based on the established cut off value.
		mRNA-1273 (n = 7)			

BNT162b2, Pfizer-BioNTech vaccine; mRNA-1273, Moderna vaccine; ChAdOx1-S, Oxford – AstraZeneca vaccine; JNJ-78436735, Johnson & Johnson vaccine; AB, antibody; BV, before vaccination; IgA, immunoglobulin A; IgG, immunoglobulin G; No., number; RBD, receptor-binding domain; pM, picomolar; BM, breast milk.

**Table 2:** Overview of vaccination studies in breastfeeding women with data on antibody secretion in breast milk, published as preprint.

Study	Number of participants	Applied vaccine(s)	Time points	IgA response in BM	IgG response in BM
Fox et al. *(USA)*	10	BNT162b2 (n = 6)	BV + 14 days after 2^nd^ dose	In 6 out of 10 undiluted post-vaccination Spike specific IgA were found.	All post-vaccination samples contained Spike-specific IgG.
		mRNA-1273 (n = 4)			
Fox et al. *(USA)*	50	BNT162b2 (n = 23)	1 week BV + 14 days after 2^nd^ dose for BNT162b2 and mRNA-1273/28 days after 1^st^ dose of JNJ-78436735	After vaccination, 71% of mRNA-1273 (mean endpoint titer of 19) and 52% of BNT162b2 (mean end point titer of 22). Of JNJ-78436735 mother milk samples 23% (endpoint titer of 15). The endpoint titer of JNJ-78436735 IgA was significantly lower than that of the mRNA-1273 vaccine group (p = 0.025).	After vaccination, 100% of mRNA-1273 (mean endpoint titer of 120) and 87% of BNT162b2 (mean endpoint titer of 180) mother milk samples contained levels of Spike-specific IgG. There was no significant difference in the mean IgG titers of both mRNA vaccine groups. both mRNA vaccine groups exhibited significantly higher specific milk IgG compared to sample of the JNJ-78436735 vaccine group. Only 38% of JNJ-78436735 samples contained levels of specific IgG (mean endpoint titer = 10; p < 0.0001).
		mRNA-1273 (n = 14)			
		JNJ-78436735 (n = 13)			
Friedman et al. *(Israel)*	10	BNT162b2	Day 7 (T1) and day 14 (T2) after the 1^st^ dose + day 7 (T3) and day 14 (T4) after 2^nd^ dose	At 14 days after the 1^st^ dose, a first significant increase in antibody titers was seen. This upward trend peaked at 7 days after the 2^nd^ dose. A slight decrease was seen in titers 14 days after the 2^nd^ dose. IgA in mother milk exhibited a potential neutralization capacity in all mothers.	At 14 days after the 1^st^ dose, a first significant increase in antibody titers was seen. This upward trend peaked 7 days after the 2^nd^ dose. A slight decrease was seen in titers 14 days after the 2^nd^ dose. Anti-spike IgG in mother milk exhibited a potential neutralization capacity in all mothers.
Golan et al. *(USA)*	48	BNT162b2 (n = 27)	BV (T1) + day of 2^nd^ dose (T2) + between 4-10 weeks after 2^nd^ dose (T3)	Twelve individuals (BNT162b2 n=7; mRNA-1237 n=5) did not have detectable levels of anti-RBD IgA at T1 and T2. There were significantly higher levels of IgA antibodies specific to SARS-CoV-2 RBD protein found in mother milk samples after the 1^st^ dose (T2). Compared to anti-RBD IgA at T2, there was no significant increase 4-10 weeks after the 2^nd^ dose.	Not Applicable
		mRNA-1273 (n = 21)			
Golan et al. *(USA)*	23	BNT162b2 (n = 14)	BV (T1) + day of 2^nd^ dose (T2) + 4 weeks after 2^nd^ dose (T3)	After the 1^st^ dose significantly higher levels of IgA AB specific to SARS-CoV-2 RBD protein in mother milk samples were found. At the day of 2^nd^ dose 17 out of 19 samples were positive for anti- SARS-CoV-2 IgA AB. Four weeks after the 2^nd^ dose 13 out of 15 samples were positive for anti-SARS-CoV-2 RBD IgA. A variation in anti-SARS-CoV-2 RBD IgA AB levels was found in samples collected from 0 to 64 days after 1^st^ doses. Four weeks after the 2^nd^ dose IgA levels largely remained stable.	Not Applicable
		mRNA-1273 (n = 9)			
Low et al. *(Singapore)*	10	BNT162b2	BV (T1) + 1-3 days after 1^st^ dose (T2) + 7-10 days after 1^st^ dose (T3) + 3-7 days after 2^nd^ dose (T4)	At 3-7 days after the 2^nd^ dose, the sharpest rise of IgA antibody production was found, with a median 374 pM. In mother milk sample of one mother, IgA was not detected in one mother 3-7 days after the 2^nd^ dose.	At 3-7 days after the 2^nd^ dose, the sharpest rise of IgG antibody production was found, with a median of 1110 pM.

BNT162b2, Pfizer-BioNTech vaccine; mRNA-1273, Moderna vaccine; ChAdOx1-S, Oxford – AstraZeneca vaccine; JNJ-78436735, Johnson & Johnson vaccine; AB, antibody; BV, before vaccination; IgA, immunoglobulin A; IgG, immunoglobulin G; No., number; RBD, receptor-binding domain; pM, picomolar; BM, breast milk.

The presence of antibodies in breast milk (mainly IgA and IgG) after vaccination against SARS-CoV-2 during lactation, have been demonstrated by several prospective cohort studies. Nearly all studies were conducted with mRNA vaccines (See **Tables 1**, **2**). Only one study included participants vaccinated with the JNJ-78436735 vaccine (48), one study included participants vaccinated with the Sinovac Biotech Ltd. Vaccine (22) and two studies included participants vaccinated with the ChAdOx1-S vaccine (19, 32).

In total, 17 published and 6 preprint studies researched the presence of IgA in breast milk after COVID-19 vaccination (18–24, 26, 28, 29, 31–33, 35, 36, 39, 40, 45, 47, 48). In general, the included studies show particularly an increase of antibody titers in breast milk after the second dose and that this is highly correlate with the levels present in the mother’s blood. An increase of anti-SARS-CoV-2 specific IgA was found in most studies one week after the first dose. In the different studies, the interval between the first and second dose of mRNA vaccines was between 21 and 35 days, however, the participants of the two studies using the adenovector-based vaccines ChAdOx1 nCoV-1, did not receive a second dose. After the intermediate timepoint between the first and second vaccine dose, a decrease of IgA was reported towards the second dose. In most studies, the highest concentrations were observed one week after the second dose (18, 20–23, 28, 29, 32, 33, 36, 39, 40, 45). Three studies compared IgA between the mRNA-based vaccines (BNT162b2, n=123, and mRNA-1273, n=45) and the adenovector-based vaccines ChAdOx1 nCoV-19 (n=44) or JNJ-78436735 (n=13) (19, 32, 48). A first study found a significant difference in the mean IgA titers in breast milk of mothers vaccinated with BNT162b2 vs. ChAdOx1 nCoV-19 (p=0.02). For mothers who received the mRNA-1273 vaccine, the BNT162b2 vaccine and the ChAdOx1 nCoV-19 vaccine (one dose) respectively, the mean antibody titers observed in milk were 0.10 (**±** SD 0.07), 0.11 (**±** SD 0.12) and 0.04 (**±** SD 0.07) (AU, Arbitrary Units) (19). Another study found that IgA levels were higher after the first dose mRNA-1273 vaccinated women compared to ChAdOx1 nCoV-19 (one dose) (p<0.0001) and BNT162b2 (p=0.002). After the second dose, no differences were observed between the mRNA-based vaccines. After the notification of severe episodes of immune thrombotic thrombocytopenia after vaccination with ChAdOx1 nCoV-19, participants did not receive a second dose. Therefore there is no information on antibody responses in breast milk available after the second dose of ChAdOx1 nCoV-19 (32). A third study compared the mRNA-based vaccines with the JNJ-78436735 vaccine. Positive levels of Spike-specific IgA, exhibiting a mean endpoint titer of 15, was found in 23% of the JNJ-78436735 recipient milk samples. Comparing to the mRNA-1273 vaccine group, this was significantly lower (p=0.025) (48).

In total, the presence of IgG in breast milk after vaccination against SARS-CoV-2 in the lactation period was researched in 17 published and 4 preprint studies (18, 19, 21, 23–26, 28–33, 35, 36, 39, 47, 48). In some milk samples, an increase of anti-SARS-CoV-2 specific IgG was found one week after the first dose and increasing towards 2 weeks after the first dose. After the second dose, also an increase of IgG antibodies was seen (18, 21, 23, 25–29, 32, 33, 36, 39). Three studies compared ant-SARS CoV-2 RDB-S1 IgG between the mRNA-based vaccines (mRNA-1273 and BNT162b2) and the adenovector-based vaccines ChAdOx1 nCoV-19 or JNJ-78436735 (19, 32, 48). In a first study, according to the type of vaccine, the mean IgG titers were different, being 0.41 (**±** SD 0.10) for mothers who received BNT162b2, 0.45 ((**±** SD 0.08) for mRNA-1273, and 0.09 (**±** SD 0.08) (AU) ChAdOx1-S (one dose).

Comparing mean of IgG of lactating women vaccinated with mRNA-1273 or BNT162b2 vs. ChAdOx1-S significant differences were found (p=0.01), but there were no differences found between those mothers vaccinated with mRNA-1273 vs. BNT162b2 (19). In another study, higher levels of IgG were induced by the mRNA-1273 vaccine and BNT162b2 vaccine compared to the ChAdOx1-S vaccine after the first dose. Compared to the ChAdOx1-S vaccine 2 weeks after the first dose, a higher percentage of samples from mRNA-based vaccines remained positive for anti-SARS-CoV-2 IgG (p<0.0001) (32). In a third study, positive levels of Spike-specific IgG were found in 100% of mRNA-1273 and 87% of BNT162b2 post-vaccine milk samples. Of these mRNA vaccine groups, there were no significant differences in mean IgG titers. Both groups, of the mRNA-1273 vaccine and BNT162b2 vaccine, exhibited significantly higher specific milk IgG compared to milk samples from mothers vaccinated with the JNJ-78436735 vaccine. Only 38% of JNJ-78436735 samples contained positive levels of specific IgG (mean endpoint titer = 10; p < 0.0001) (48).

In addition, the presence of binding neutralizing antibodies was revealed in two studies evaluating the immunogenicity of mRNA-based vaccines (24, 36). These data may indicate that breast milk has the potential to add to infant protection by passively transferred antibodies through breast milk.

### Excretion of Other Immunological Factors in Breast Milk

Only one manuscript reported on the excretion of other immunological factors than antibodies. The study included 14 women, all receiving the BNT162b2 vaccine. The study showed that vaccination is not only able to increase the amount of antibodies in breast milk, but also induces spike-reactive CD4+ T cells in breast milk, especially after the second dose of the BNT162b2 vaccine. These spike-reactive CD4+ T cells may have a protective function in the upper respiratory tract of infants (37).

### Impact of Vaccination on Breast Milk Production

Five manuscripts report on the impact of vaccination on breast milk production and included a total of 11,586 lactating women receiving a COVID-19 vaccine (38, 42, 43, 45). These studies report on 4,399 women vaccinated with the BNT162b2 vaccine, 2,669 with the mRNA-1273, 23 with the JNJ-78436735 and for 40 the vaccine type was not specified (42, 43, 45). One of these studies vaccinated 4,445 women with either the mRNA-1273 or the BNT162b2 vaccine. This was not specified (38).

In a first study (n = 180), a temporary reduction in breast milk supply was reported by some women after vaccination with a COVID-19 mRNA vaccine (71% BNT162b2, 29% mRNA-1273). A decrease in milk production after the BNT162b2 vaccine was reported by 7.3% (9/126) and 8.0% (9/123) women after the first and second dose, respectively. The percentage of women reporting a decrease in milk production after the mRNA-1273 vaccine was 11.5% (6/52) after the first dose and 23.4% (11/52) after the second dose. The difference between the BNT162b2 vaccine and the mRNA-1273 was statistically significant (p<0.05). Milk supply returned to normal within three days in all cases. In contrast, an increase in milk supply was reported by some women. More production was reported after the first dose by 3.3% (4/126) of mothers who received the BNT162b2 vaccine, but was not reported by mothers receiving a first dose of the mRNA-1273 vaccine. After the second dose, 3.6% (4/123) of mothers who received the BNT162b2 vaccine reported an increase in milk supply, and 6.4% (3/52) of mothers who received the mRNA-1273 vaccine. Finally, a milk color change to blue-green color was reported by 3 mothers after vaccine administration (2/126, 8.0%, BNT162b2; 1/52, 7.1%, mRNA-1273) after the administration of the first dose and by 2 mothers (1/123, 4.0%, BNT162b2; 1/52, 6.2%, mRNA-1273) after the second dose (43).

In a second study, 4,455 breastfeeding mothers who received either the BNT162b2 vaccine or the mRNA-1273 vaccine filled in an online survey. An increase in milk supply was reported by 3.9% of mothers and a decrease was reported by 6.0% of mothers (38). In a third study of 6,815 lactating women, 339 participants reported a decreased milk supply no longer than 24 hours after the first dose (5.0%) and 434 participants after the second dose (7.2%) (42). In another, relatively small study with 48 mothers, 2 mothers reported a slight decrease in milk production in the first 24-72 hours after the first and second dose (45).

An interruption of breastfeeding after the first dose by 155 of 6,815 participants (2.3%) and 130 of 6,056 individuals after the second dose (2.2%) was reported by Kachikis et al. (2021). Whether the breastfeeding interruption was a deliberate choice of the mother, imposed upon from the health care provider or a consequence of decreased production was not specified (42).

One study looked at milk production after receiving the BNT162b2 vaccine (n = 88). A change in milk supply was reported by one woman, increase or decrease was not specified. One woman reported a transient bluish-green color of her breast milk after her first vaccine dose. This was not reported after her second dose (44).

## Discussion

### Main Findings of the Review

We reviewed the literature on the safety of COVID-19 vaccination during lactation in women and neonates. Subsequently, we summarized the effects of COVID-19 vaccination during lactation on the excretion of COVID-19 vaccine components in breast milk, the excretion of immunologic factors in breast milk and on the production of breast milk. In general, currently available data point towards a reassuring safety profile of COVID-19 vaccination during lactation with comparable side-effects in lactating compared to pregnant, non-pregnant and non-lactating women of childbearing age. While vaccine components are barely or not detectable in breast milk, most studies report the presence of anti-SARS-CoV-2 IgA and IgG in breast milk at several timepoints post vaccination, up to 8 weeks after the second vaccine dose. Additionally, a large proportion of the antibodies in breast milk exhibits neutralizing capacity against the virus offering potential additional protection to the nursing infant. The impact on milk supply appears to remain very limited.

### Combining the Reported Study Outcomes

Since neonates are born with an immature immune system, they rely on the transfer of antibodies *via* the placenta and breast milk for their protection during the first vulnerable months and years of life. The mechanisms by which the antibodies in breast milk provide protection to the neonate still remain unclear (15). At the moment of publication, worldwide, 47 out of 224 countries recommend COVID-19 vaccination for some or all lactating women whereas 77 countries state that lactating women can receive, may receive or can choose to receive the vaccine. In 8 countries, only certain groups of lactating people (e.g. health care professionals, women with underlying conditions) can or may choose to receive the vaccine. One country state that lactating women should not receive the vaccine, with certain exceptions and 23 countries do not recommend COVID-19 vaccination for lactating women. For 68 countries, there is currently no information on their policy regarding COVID-19 vaccination during lactation (10).

Despite the fact that the strategy of vaccinating lactating women with COVID-19 vaccines is frequently recommended on a global level, none of the COVID-19 vaccines currently authorized or in phase 3 have been trialed for women who are breastfeeding. Since COVID-19 is a new disease, it is important to consider whether vaccination during lactation is safe and effective. The safety of administrating inactivated vaccines to lactating women was already shown for other vaccine-preventable infectious diseases (49) and also sIgA and IgG after postpartum vaccination against pertussis and influenza are proven to be secreted into breast milk (15).

Three studies, all using mRNA-based vaccines, focused on the safety and side-effects of COVID-19-vaccination in lactating women (42, 43, 45). Among all participants, the most common described side-effects were pain at the injection site, fatigue, chills, headache, muscle/body aches, fever and vomiting. These side-effects are similar to the ones seen in the general population (46).

The excretion of COVID-19 vaccine components in breast milk was investigated in two studies (17, 41). Although these studies were small, it is reassuring that no or very low concentrations of mRNA were detected (17, 41). If low concentrations of mRNA reach the breastfed infant through breast milk, it is very likely that there will be no uptake by the gastrointestinal system. The small amount of polyethylene glycol-2000 (PEG-2000) in the BNT162b2 vaccine is not found in breast milk. This is important to know, since PEG-2000 can cause anaphylaxis in very rare cases (45). No studies were performed on the excretion of COVID-19-vaccine components in breast milk after vaccination with any other type of COVID-19 vaccine.

Multiple studies showed that sIgA and IgG against the SARS-CoV-2 spike protein are present in breast milk after COVID-19 mRNA vaccination (20, 21, 26, 37, 50, 51). These studies show that the antibody titers (sIgA and IgG) in breast milk mainly increase after a second dose and that they are strongly correlated with the antibody levels present in the mother’s blood. In most published studies, antibody levels are measured relatively shortly after vaccination, i.e. within 2 to 8 weeks after the second dose. At the time of publication, most studies focused on measuring antibody levels until 2 to 8 weeks after the second dose. Long-term results on antibody levels after vaccination are not published yet.

Nearly all studies were conducted with mRNA vaccines. Only one study was conducted with the JNJ-78436735 vaccine (48), one study was conducted with the Sinovac Biotech Ltd. vaccine (22) and two studies were conducted with the ChAdOx1-S vaccine (19, 32).

Five studies looked at the effect of COVID-19 vaccination on milk production (38, 42–45). Only a few women reported a temporary reduction in breast milk supply. Milk supply returned to normal within one to three days. Some women reported an increase in milk supply.

### Strengths and Limitations of the Review

To the best of our knowledge, this is the most complete and extensive review on the effects of COVID-19 vaccines when administered to lactating women. The insights of this review are important for policy makers that can adapt guidelines and inform women on whether to take the vaccine or not during lactation as vaccination during lactation could result in clinically relevant immunological factors in breast milk and therefore offer additional protection to the infant.

Despite specific selection criteria of the studies included in this review, differences in vaccine schedules, sample collection timepoints, sample processing and data monitoring complicated head-to-head comparisons between studies and performing a meta-analysis was not possible. This also means that at this point, it is not possible to compare the different vaccine platforms used in lactating women. Also, since COVID-19 vaccination is a rapidly evolving field, pre-prints or publications might have been missed when published between submission and publication of the manuscript.

The review did not include studies on COVID-19 vaccination of pregnant women and the effect of antibodies in their breast milk.

### Recommendations for Future Practice and Research

Side-effects for both mother and infant were researched in only four studies. Sample sizes were however large enough to provide a first indication of safety. However, the effect of vaccination on the milk production was studied based on (retrospective) reporting by the mother, which could have led to a recall bias. Quantitative research into the effect on milk supply could give more insights and could be used for further recommendations about vaccination during lactation. Additionally, it is essential that research on specific breastfeeding related side-effects is performed. This includes for example breast engorgement or the development of mastitis. These are currently often not taken into account. It would also be of interest to know whether changing the vaccine administration place (i.e. leg instead of arm) would lead to less breastfeeding related side-effects.

At this point, most studies researched the excretion of antibodies after COVID-19 vaccination with an mRNA-based vaccine. The two studies that used the adenovector-based vaccine ChadOx1-S were stopped after administration of the first dose. At the moment, in most countries, adenovector-based vaccines are no longer used in women of childbearing age, but there are other vaccines developed with this platform for other diseases, such as Ebola. The information on the effect of adenovector-based vaccines in lactating women could be interesting to extrapolate to other vaccines in development that have the potential to be used in women of fertile age. Research on the safety, side-effects and excretion of antibodies after a full vaccine schedule including booster vaccination is therefore adamant. Worldwide the booster vaccination (a third vaccination after mRNA-based vaccines or the ChadOx1-S vaccine or a second vaccination after the JNJ-78436735 vaccine), is being recommended (52, 53). At the moment there are no published studies on the effects of COVID-19 booster vaccination in lactating women. Finally, a qualitative analysis of the subclass, glycosylation profile and functional properties of vaccine-induced antibodies would be of interest to enlighten the immunology during breastfeeding. This could be completed along more research into cellular and humoral immune responses during lactation. Lastly, one of the main questions are whether these immunological factors excreted into breast milk also have protective effects for the infant.

Additionally, as the WHO and UNICEF recommend 6 months exclusive breastfeeding and afterwards until the age of 2 years in combination with complementary foods (13, 14), more knowledge on the effects of vaccination on the long term is indispensable. Most studies focused on breast milk analysis up to 2 or 8 weeks after the second dose. Therefore, there is a need for follow-up studies taking samples with longer time intervals and longer follow-up.

Safety of vaccination during lactating period need to be assessed at early stages of product development. In order to achieve this, vaccine manufacturers and regulators must work closely with specialists in lactation, infectious diseases and public health experts in order to improve maternal and infant health and to build confidence in vaccines. Breastfeeding women therefore need to be included in clinical trials and the need for appropriate safety data is critical.

## Conclusion

There is no evidence that the administration of a COVID-19 vaccine poses an additional risk to the breastfeeding woman or the breastfed infant. Data on safety of vaccines against SARS-CoV-2 virus indicate no severe vaccine-related local and systemic reactions, both after first and second dose. Milk supply data after vaccination indicate that some women report a temporary reduction in milk supply, without a long-term effect and milk supply returned to normal within a few days whereas other women reported a stimulation of breast milk production. All prospective cohort studies have demonstrated the presence of antibodies (mainly sIgA and IgG) in breast milk of nursing mothers vaccinated against SARS-CoV-2. Nearly all studies were conducted with mRNA vaccines. These studies mainly showed that the antibody titers in breast milk mainly increase after the second dose and are associated with the levels present in the mother’s blood.

After vaccination of the mother, antibodies appear in the milk, which could better protect the infant against COVID-19. Professional associations and government health authorities should recommend offering COVID-19 vaccines to breastfeeding women, as the potential benefits of maternal vaccination while breastfeeding outweigh the theoretical risks.

## Data Availability Statement

The original contributions presented in the study are included in the article/supplementary material. Further inquiries can be directed to the corresponding author.

## Author Contributions

JM and ET conducted the review. LW, LB, and KM read the review and gave new input and comments. All authors contributed to the article and approved the submitted version.

## Funding

Funding was received by The Mustela Foundation. JM is attached to the Vrije Universiteit Brussel, Belgium, as a volunteer researcher. LW, LB, and KM are supported by the University of Antwerp, Belgium. ET is supported by the Vrije Universiteit Brussel, Belgium.

## Conflict of Interest

All authors have completed the ICMIJE uniform disclosure form at www.icmje.org/disclosure-of-interest/. ET received a fee for personal consulting for expertise considering smoking cessation counselling in the community pharmacy from Johnson and Johnson.

The remaining authors declare that the research was conducted in the absence of any commercial or financial relationships that could be construed as a potential conflict of interest.

## Publisher’s Note

All claims expressed in this article are solely those of the authors and do not necessarily represent those of their affiliated organizations, or those of the publisher, the editors and the reviewers. Any product that may be evaluated in this article, or claim that may be made by its manufacturer, is not guaranteed or endorsed by the publisher.

## APPENDIX 1: Detailed search strategy of the systematic review

A. Define text words & synonyms for the text words

1. (COVID-19* or SARS-CoV-2* or corona disease* or COVID*).af.
2. (breastfeeding* or lactation* or breast milk*).af.
3. (postpartum* or postnatal period* or puerperium*).af.
4. (pregnancy*).af.

B. Perform test searches – I

1 AND 2
1 AND 3
1 AND 4
Limit to following languages: German, Dutch, English, French & Spanish
5, 6, 7 AND 8 AND 2020/12/01 to 2021/12.31.date

C. Identify “controlled vocabulary” (keywords) used for the indexing of databases (MeSH)

“COVID-19 Vaccines” or “COVID-19” or “SARS-CoV-2” (Mesh)
“Lactation” or “Breast Feeding” (Mesh)
“Postpartum Period” (Mesh)
“Pregnancy” (Mesh)

D. Perform test searches – II

10 AND 11
10 AND 12
10 AND 13
Limit to following languages: German, Dutch, English, French & Spanish
14, 15 AND 16 AND 2020/12/01 to 2021/12/31.date

E. Remove duplicates

## References

[1] World Health Organization . Who-Director-General-s-Opening-Remarks-at-the-Media-Briefing-on-Covid-19—11-March-2020 (2020). Available at: https://www.who.int/director-general/speeches/detail/who-director-general-s-opening-remarks-at-the-media-briefing-on-covid-19—11-march-2020.

[2] Guan W Ni Z Hu Y Liang W Ou C He J . Clinical Characteristics of Coronavirus Disease 2019 in China. N Engl J Med (2020) 382:1708–20. doi: 10.1056/NEJMoa2002032 PMC7092819 32109013

[3] European Commission . Coronavirus: Commission Lists Key Steps for Effective Vaccination Strategies and Vaccines Deployment (2020). Available at: https://ec.europa.eu/commission/presscorner/detail/en/ip_20_1903.

[4] Federal Government Belgium . Coronavirus COVID-19 Vaccination. Available at: https://www.info-coronavirus.be/en/vaccination/.

[5] Heinz FX Stiasny K . Distinguishing Features of Current COVID-19 Vaccines: Knowns and Unknowns of Antigen Presentation and Modes of Action. NPJ Vaccines (2021) 6:1–13. doi: 10.1038/s41541-021-00369-6 34400651 PMC8368295

[6] Hare H Womersley K . Why Were Breastfeeding Women in the UK Denied the Covid-19 Vaccine? BMJ (2021) 372 January:5–6. doi: 10.1136/bmj.n4 33402337

[7] Merewood A Bode L Davanzo R Perez-Escamilla R . Breastfeed or be Vaccinated—An Unreasonable Default Recommendation. Lancet (2021) 397:578. doi: 10.1016/S0140-6736(21)00197-5 33549167 PMC9753227

[8] European Medicines Agency . International-Cooperation-Align-Approaches-Regulation-Covid-19-Vaccines-Medicines @ Www.Ema.Europa.Eu. Available at: https://www.ema.europa.eu/en/news/international-cooperation-align-approaches-regulation-covid-19-vaccines-medicines.

[9] Royal College of Obstetricians & Gynaecologists . COVID-19 Vaccines, Pregnancy and Breastfeeding @. Available at: www.rcog.org.uk.

[10] World Health Organization . Covid-19 Vaccine Policies on Lactation. Available at: https://www.comitglobal.org/explore/public-health-authorities/lactation.

[11] CDC . COVID-19 Information About COVID-19 Vaccines for People Who Are Pregnant or Breastfeeding Limited Data Are Available About the Safety of COVID-19 Vaccines for People Who Are Pregnant Follow Recommendations to Prevent the Spread of COVID-19 After Vaccinati. USA: CDC (2021). pp. 2019–22.

[12] Department of Health & Social Care UK . Joint-Committee-on-Vaccination-and-Immunisation-Advice-on-Priority-Groups-for-Covid-19-Vaccination-30-December-2020 @ Www.Gov.Uk. Available at: https://www.gov.uk/government/publications/priority-groups-for-coronavirus-covid-19-vaccination-advice-from-the-jcvi-30-december-2020/joint-committee-on-vaccination-and-immunisation-advice-on-priority-groups-for-covid-19-vaccination-30-december-2020.

[13] World Health organization . Infant-And-Young-Child-Feeding. Available at: http://www.who.int/en/news-room/fact-sheets/detail/infant-and-young-child-feeding.

[14] UNICEF . Breastfeeding And Family-Friendly Policies An Evidence Brief Breastfeeding And Family-Friendly Policies. In: An Evidence Brief Griswold M Palmquist A . New York, USA (2019).

[15] Atyeo C Alter G . The Multifaceted Roles of Breast Milk Antibodies. Cell (2021) 184:1486–99. doi: 10.1016/j.cell.2021.02.031 33740451

[16] Hutton B Salanti G Caldwell DM Chaimani A Schmid CH Cameron C . The PRISMA Extension Statement for Reporting of Systematic Reviews Incorporating Network Meta-Analyses of Health Care Interventions: Checklist and Explanations. Ann Intern Med (2015) 162:777–84. doi: 10.7326/M14-2385 26030634

[17] Golan Y Prahl M Cassidy A Lin CY Ahituv N Flaherman VJ . COVID-19 mRNA Vaccine is Not Detected in Human Milk. Pre-print medRxiv (2021), 1–8. doi: 10.1101/2021.03.05.21252998

[18] Kelly JC Carter EB Raghuraman N Nolan LS Gong Q Lewis AN . Anti–SARS-CoV-2 Antibodies Induced in Breast Milk After Pfizer-BioNTech/BNT162b2 Vaccination. Am J Obstet Gynecol (2021) 225:101–3. doi: 10.1016/j.ajog.2021.03.031 PMC8062573 33798480

[19] Lechosa-Muñiz C Paz-Zulueta M Mendez-Legaza JM Irure-Ventura J González RC Montes JC . Induction of Sars-Cov-2-Specific Igg and Iga in Serum and Milk With Different Sars-Cov-2 Vaccines in Breastfeeding Women: A Cross-Sectional Study in Northern Spain. Int J Environ Res Public Health (2021) 18:1–12. doi: 10.3390/ijerph18168831 PMC8393848 34444579

[20] Golan Y Prahl M Cassidy A Wu AHB Jigmeddagva U Lin CY . Immune Response During Lactation After Anti-SARS-CoV2 mRNA Vaccine. medRxiv (2021) 94143:2021.03.09.21253241. doi: 10.1101/2021.03.09.21253241v1

[21] Baird JK Jensen SM Urba WJ Fox BA Baird JR . SARS-CoV-2 Antibodies Detected in Human Breast Milk Post-Vaccination. medRxiv (2021) 2021.02.23.21252328. doi: 10.1101/2021.02.23.21252328 PMC8685565 34297643

[22] Calil VMLT Palmeira P Zheng Y Krebs VLJ de Carvalho WB Carneiro-Sampaio M . Coronavac can Induce the Production of Anti-Sars-Cov-2 Iga Antibodies in Human Milk. Clinics (2021) 76:1–2. doi: 10.6061/clinics/2021/e3185 PMC8221553 34190859

[23] Charepe N Gonçalves J Juliano AM Lopes DG Canhão H Soares H . COVID-19 mRNA Vaccine and Antibody Response in Lactating Women: A Prospective Cohort Study. BMC Pregnancy Childbirth (2021) 21:1–9. doi: 10.1186/s12884-021-04051-6 34535094 PMC8447894

[24] Collier ARY McMahan K Yu J Tostanoski LH Aguayo R Ansel J . Immunogenicity of COVID-19 mRNA Vaccines in Pregnant and Lactating Women. JAMA J Am Med Assoc (2021) 325:2370–80. doi: 10.1001/jama.2021.7563 PMC8120446 33983379

[25] Esteve-Palau E Gonzalez-Cuevas A Guerrero ME Garcia-Terol C Alvarez MC Casadevall D . Quantification of Specific Antibodies Against SARS-CoV-2 in Breast Milk of Lactating Women Vaccinated With an mRNA Vaccine. JAMA Netw Open (2021) 4:18–20. doi: 10.1001/jamanetworkopen.2021.20575 PMC8358734 34379127

[26] Gray KJ Bordt EA Atyeo C Deriso E Akinwunmi B Young N . Coronavirus Disease 2019 Vaccine Response in Pregnant and Lactating Women: A Cohort Study. medRxiv (2021) 2021.03.07.21253094. doi: 10.1016/j.ajog.2021.03.023 PMC7997025 33775692

[27] Guida M Terracciano D Cennamo M Aiello F La Civita E Esposito G . COVID-19 Vaccine Mrnabnt162b2 Elicits Human Antibody Response in Milk of Breastfeeding Women. Vaccines (2021) 9:3–9. doi: 10.3390/vaccines9070785 PMC8310008 34358201

[28] Jakuszko K Kościelska-Kasprzak K Żabińska M Bartoszek D Poznański P Rukasz D . Immune Response to Vaccination Against Covid-19 in Breastfeeding Health Workers. Vaccines (2021) 9:1–10. doi: 10.3390/vaccines9060663 PMC8235492 34204501

[29] Low JM Gu Y Ng MSF Amin Z Lee LY Ng YPM . Codominant IgG and IgA Expression With Minimal Vaccine mRNA in Milk of BNT162b2 Vaccinees. NPJ Vaccines (2021) 6:1–8. doi: 10.1038/s41541-021-00370-z 34413319 PMC8376902

[30] Nir O Schwartz A Toussia-Cohen S Leibovitch L Strauss T Asraf K . Maternal-Neonatal Transfer of SARS-CoV-2 Immunoglobulin G Antibodies Among Parturient Women Treated With BNT162b2 Messenger RNA Vaccine During Pregnancy. Am J Obstet Gynecol MFM (2022) 4:100492. doi: 10.1016/j.ajogmf.2021.100492 34547533 PMC8451978

[31] Romero Ramírez DS Lara Pérez MM Carretero Pérez M Suárez Hernández MI Martín Pulido S Pera Villacampa L . SARS-CoV-2 Antibodies in Breast Milk After Vaccination. Pediatrics (2021) 148:e2021052286. doi: 10.1542/peds.2021-052286 34408089

[32] Selma-Royo M Bauerl C Mena-Tudela D Aguilar-Camprubi L Perez-Cano FJ Parra-Llorca A . Anti-Sars-Cov-2 IgA and IgG in Human Milk After Vaccination Is Dependent on Vaccine Type and Previous Sars-Cov-2 Exposure A Longitudinal Study. medRxiv (2021) 2021.05.20.21257512. doi: 10.1101/2021.05.20.21257512v1 PMC9022055 35449030

[33] Valcarce V Stafford LS Neu J Cacho N Parker L Mueller M . Detection of SARS-CoV-2-Specific IgA in the Human Milk of COVID-19 Vaccinated Lactating Health Care Workers. Breastfeed Med (2021) XX Xx:1–6. doi: 10.1089/bfm.2021.0122 34427487

[34] Fox A DeCarlo C Yang X Norris C Powell RL . Comparative Profiles of SARS-CoV-2 Spike-Specific Milk Antibodies Elicited by COVID-19 Vaccines Currently Authorized in the USA. medRxiv (2021), 1–19. doi: 10.1101/2021.07.19.21260794 35675683

[35] Fox A Norris C Amanat F Zolla-Pazner S Powell RL . The Vaccine-Elicited Immunoglobulin Profile in Milk After COVID-19 mRNA-Based Vaccination is IgG Dominant and Lacks Secretory Antibodies. medRxiv (2021), 1–14. doi: 10.1101/2021.03.22.21253831

[36] Rosenberg-Friedman M Kigel A Bahar Y Yogev Y Dror Y Lubetzky R . BNT162b2 COVID-19 mRNA Vaccine Elicits a Rapid and Synchronized Antibody Response in Blood and Milk of Breastfeeding Women. medRxiv (2021) 2021.03.06.21252603. doi: 10.1101/2021.03.06.21252603v1 PMC8553805 34711825

[37] Gonçalves J Juliano AM Charepe N Alenquer M Athayde D Ferreira F . Non-Neutralizing Secretory IgA and T Cells Targeting SARS-CoV-2 Spike Protein are Transferred to the Breastmilk Upon BNT162b2 Vaccination. medRxiv (2021) 2021.05.03.21256416. doi: 10.1016/j.xcrm.2021.100468 PMC8636305 34873588

[38] McLaurin-Jiang S Garner CD Krutsch K Hale TW . Maternal and Child Symptoms Following COVID-19 Vaccination Among Breastfeeding Mothers. Breastfeed Med (2021) 16:10–1. doi: 10.1089/bfm.2021.0079 34171971

[39] Perl SH Uzan-Yulzari A Klainer H Asiskovich L Youngster M Rinott E . SARS-CoV-2-Specific Antibodies in Breast Milk After COVID-19 Vaccination of Breastfeeding WOmen. JAMA J Am Med Assoc (2021) 325(19):2013–4. doi: 10.1001/jama.2021.5782 PMC8042567 33843975

[40] Juncker HG Mulleners SJ van Gils MJ Bijl TPL de Groot CJM Pajkrt D . Comparison of SARS-CoV-2-Specific Antibodies in Human Milk After mRNA-Based COVID-19 Vaccination and Infection. Vaccines (2021) 00(0):1–11. doi: 10.3390/vaccines9121475 PMC8706455 34960222

[41] Low JM Gu Y Shu Feng Ng M Amin Z Ye Lee L Peng Mei Ng Y . BNT162b2 Vaccination Induces SARS-CoV-2 Specific Antibody Secretion Into Human Milk With Minimal Transfer of Vaccine mRNA. medRxiv (2021) 2021:4. doi: 10.1101/2021.04.27.21256151

[42] Kachikis A Englund JA Singleton M Covelli I Drake AL Eckert LO . Short-Term Reactions Among Pregnant and Lactating Individuals in the First Wave of the COVID-19 Vaccine Rollout. JAMA Netw Open (2021) 4:9–13. doi: 10.1001/jamanetworkopen.2021.21310 PMC8371565 34402893

[43] Bertrand K Honerkamp-Smoth G Chambers C . Maternal and Child Outcomes Reported by Breastfeeding Women Following mRNA COVID- 19 Vaccination. medRxiv (2021), 1–8. doi: 10.1101/2021.04.21.21255841 PMC8563461 34492204

[44] Low JM Lee LY Peng Mei Ng Y Zhong Y Amin Z . Breastfeeding Mother-Child Clinical Outcomes After COVID-19 Vaccination. medRxiv (2021), 1–17. doi: 10.1101/2021.06.19.21258892 34713745

[45] Golan Y Prahl M Cassidy AG Gay C Wu AHB Jigmeddagva U . COVID-19 mRNA Vaccination in Lactation: Assessment of Adverse Effects and Transfer of Anti-SARS-CoV2 Antibodies From Mother to Child. medRxiv Prepr Serv Heal Sci (2021), 1–41. doi: 10.1101/2021.03.09.21253241

[46] Prevention C for DC and Possible Side Effects After Getting a COVID-19 Vaccine (2021). Available at: https://www.cdc.gov/coronavirus/2019-ncov/vaccines/expect/after.html.

[47] Schwartz A Nir O Toussia-Cohen S . Presence of SARS-CoV-2 Antibodies in Lactating Women and Their Infants Following BNT162b2 Messenger RNA Vaccine. Am J Obstet Gynecol (2021) 577–9. doi: 10.1016/j.ajog.2021.07.016 PMC8327579 34352250

[48] Fox A Norris C Amanat F Zolla-Pazner S Powell RL . The Vaccine-Elicited Immunoglobulin Profile in Milk After COVID-19 mRNA-Based Vaccination Is IgG-Dominant and Lacks Secretory Antibodies. medRxiv (2021), 1–14. doi: 10.1101/2021.03.22.21253831

[49] Prevention C for DC and. Vaccination Safety for Breastfeeding Mothers. Available at: https://www.cdc.gov/breastfeeding/breastfeeding-special-circumstances/vaccinations-medications-drugs/vaccinations.html.

[50] Atyeo C DeRiso EA Davis C Bordt EA DeGuzman RM Shook LL . COVID-19 mRNA Vaccines Drive Differential Fc-Functional Profiles in Pregnant, Lactating, and non-Pregnant Women. bioRxiv (2021) 2021:4. doi: 10.1101/2021.04.04.438404v1 PMC9067624 34664972

[51] Polack FP Thomas SJ Kitchin N Absalon J Gurtman A Lockhart S . Safety and Efficacy of the BNT162b2 mRNA Covid-19 Vaccine. N Engl J Med (2020) 383:2603–15. doi: 10.1056/NEJMoa2034577 PMC7745181 33301246

[52] World Health Organisation . Interim Statement on Booster Doses for COVID-19 Vaccination (2021). Available at: https://www.who.int/news/item/22-12-2021-interim-statement-on-booster-doses-for-covid-19-vaccination—update-22-december-2021.

[53] Richterman A Scott J Cevik M . Covid-19 Vaccines, Immunity, and Boosters. Bmj (2021) 375:n3105. doi: 10.1136/bmj.n3105 34930779

